# The Emergence of Population Health in US Academic Medicine: A Qualitative Assessment

**DOI:** 10.1001/jamanetworkopen.2019.2200

**Published:** 2019-04-12

**Authors:** Marc N. Gourevitch, Lesley H. Curtis, Maureen S. Durkin, Angela Fagerlin, Annetine C. Gelijns, Richard Platt, Belinda M. Reininger, Judith Wylie-Rosett, Katherine Jones, William M. Tierney

**Affiliations:** 1 Department of Population Health, New York University School of Medicine, New York; 2 Department of Population Health Sciences, Duke University School of Medicine, Durham, North Carolina; 3 Department of Population Health, University of Wisconsin School of Medicine and Public Health, Madison; 4 Department of Population Health Sciences, University of Utah School of Medicine, Salt Lake City; 5 Department of Population Health Science and Policy, Icahn School of Medicine at Mount Sinai, New York, New York; 6 Department of Population Medicine, Harvard Medical School, Harvard Pilgrim Health Care Institute, Boston, Massachusetts; 7 Department of Population Health and Behavioral Sciences, University of Texas Rio Grande Valley School of Medicine, Edinburg; 8 Department of Epidemiology and Population Health, Albert Einstein College of Medicine, Montefiore Health System, Bronx, New York; 9 Design Institute for Health, Dell Medical School, The University of Texas at Austin; 10 Department of Population Health, Dell Medical School, The University of Texas at Austin

## Abstract

**Importance:**

In response to rapidly growing interest in population health, academic medical centers are launching department-level initiatives that focus on this evolving discipline. This trend, with its potential to extend the scope of academic medicine, has not been well characterized.

**Objective:**

To describe the emergence of departments of population health at academic medical centers in the United States, including shared areas of focus, opportunities, and challenges.

**Design, Setting, and Participants:**

This qualitative study was based on a structured in-person convening of a working group of chairs of population health–oriented departments on November 13 and 14, 2017, complemented by a survey of core characteristics of these and additional departments identified through web-based review of US academic medical centers. United States medical school departments with the word *population* in their name were included. Centers, institutes, and schools were not included.

**Main Outcomes and Measures:**

Departments were characterized by year of origin, areas of focus, organizational structure, faculty size, teaching programs, and service engagement. Opportunities and challenges faced by these emerging departments were grouped thematically and described.

**Results:**

Eight of 9 population health–oriented departments in the working group were launched in the last 6 years. The 9 departments had 5 to 97 full-time faculty. Despite varied organizational structures, all addressed essential areas of focus spanning the missions of research, education, and service. Departments varied significantly in their relationships with the delivery of clinical care, but all engaged in practice-based and/or community collaboration. Common attributes include core attention to population health–oriented research methods across disciplines, emphasis on applied research in frontline settings, strong commitment to partnership, interest in engaging other sectors, and focus on improving health equity. Tensions included defining boundaries with other academic units with overlapping areas of focus, identifying sources of sustainable extramural funding, and facilitating the interface between research and health system operations.

**Conclusions and Relevance:**

Departments addressing population health are emerging rapidly in academic medical centers. In supporting this new framing, academic medicine affirms and strengthens its commitment to advancing population health and health equity, to improving the quality and effectiveness of care, and to upholding the social mission of medicine.

## Key Points

**Question:**

How are academic medical centers responding to the sharp rise in interest in improving the health of populations?

**Findings:**

This qualitative study found an emerging trend in which new academic departments are launching in US medical schools to serve as home to research, education, and service efforts to improve population health and health equity. Shared approaches include a strong focus on methods and applied research, partnerships, and working across sectors.

**Meaning:**

Population health is becoming the framework by which academic medicine is working to bridge the worlds of medicine and public health and of delivery system and community.

## Teaser

This qualitative study describes the emergence of departments of population health at US academic medical centers, including shared areas of focus, opportunities, and challenges.

## Introduction

Much attention has been focused in recent years on improving population health. This emphasis marks an acknowledgment that the poor performance of the United States on many indicators of health has persisted despite US leadership in many aspects of health care delivery and that other factors, like social determinants of health, must receive greater attention if national health goals and health care cost-savings goals are to be achieved.^1^ Further, improving population health aligns with language introduced through the triple-aim framework^2^ and the accompanying shift toward a value-based health care paradigm, in which accountable care and accountable health communities figure prominently. Finally, the focus on population health reflects advances in addressing root causes of health and disease and in identifying and testing sector- and discipline-bridging approaches to health promotion and disease prevention to improve health and health equity.

Most of the core concepts underlying this shift are not new. Social and economic determinants of health and disease have been characterized for centuries.^3^ Advances in public health at the whole-population level have yielded enormous reductions in the impact of infectious diseases, tobacco, injuries, and diet. While much of this work historically has been accomplished with only minimal contributions from health care and academic medicine, these sectors now command an enormous concentration of health-related resources. The Patient Protection and Affordable Care Act, intended in part to rein in rising health care costs, included community benefit language to spur nonprofit hospitals (including most academic medical centers [AMCs]) to focus on communities’ health needs.^4^ Rising attention to pervasive health disparities and their fundamental causes has further elevated the conversation about societal drivers of health and the value of shifting deliberately to a more upstream focus.^5,6^ An emerging group of population health–oriented initiatives within academic medicine is seeking to transcend the traditional gulf between medicine and public health—engaging all sectors, including health care and public health—in understanding and improving the health of populations.

The term *population health* has 2 common complementary but not interchangeable uses.^7^ Often, it refers to the health and determinants of health among a group of patients receiving health care from a particular health care institution or system. Increasingly, health care delivery systems, including those aligned with AMCs, have operational initiatives focused specifically on managing population health from a clinical perspective. Yet *population health* also refers to the health and determinants of health of persons residing in a particular region or community, irrespective of whether or where they receive health care. This comprehensive view of population health considers health care one among many core drivers of population health status, maintains a focus on health equity,^8^ and is a principal focus of new departments, centers, and institutes emerging at schools of medicine across the county.

Academic medicine has never had a uniform approach to addressing the core disciplines that contribute to population health science, ranging from social science, epidemiology, and biostatistics to prevention science and community health. Rather, medical schools have organized these themes to align with local strengths and interests, often in departments with names that include 1 or more of the following words: *prevention*, *community*, *social*, *epidemiology*, *biostatistics*, *quantitative science*, *public health*, and *health sciences*. This proliferation of titles, areas of focus, and structures has hampered the coherence and impact of population-oriented thinking within academic medicine.

Recently, spurred in part by the groundswell of attention to population health, departments and other organizational structures with a stated population focus have arisen in US medical schools. These departments, although still varied in structure and thematic focus, reflect growing recognition of the important role academic medicine has in advancing total population health. We present an overview of the emergence of this new field within academic medicine and guidance for its growth and maturation.

## Methods

We adopted 2 approaches to acquiring the data that underlie this article. First, a working group of chairs of 9 population-focused medical school departments convened to discuss the growth of the field and shared challenges and opportunities. Second, we conducted a horizon scan of potentially related initiatives in medical schools across the county. The University of Texas at Austin Institutional Review Board determined that this study was not human participants research and waived informed consent. This qualitative study is reported using Consolidated Criteria for Reporting Qualitative Research (COREQ) reporting guideline.

To help inform the launch of a Department of Population Health at the new Dell Medical School at the University of Texas at Austin, 1 of us (W.M.T.) initiated a series of conference calls with 9 leaders (department chairs or their representatives) of population-focused medical school departments, followed by an in-person meeting in Austin, Texas, on November 13 and 14, 2017. Participants were selected to represent all such departments known to the convener at the time and were approached via email. The 9 included departments were the Department of Population Health, New York University School of Medicine, New York; Department of Population Health Sciences, Duke University School of Medicine, Durham, North Carolina; Department of Population Health, University of Wisconsin School of Medicine and Public Health, Madison; Department of Population Health Sciences, University of Utah School of Medicine, Salt Lake City; Department of Population Health Science and Policy, Icahn School of Medicine at Mount Sinai, New York, New York; Department of Population Medicine, Harvard Medical School, Harvard Pilgrim Health Care Institute, Boston, Massachusetts; Department of Population Health and Behavioral Sciences, University of Texas Rio Grande Valley School of Medicine, Edinburg; Department of Epidemiology and Population Health, Albert Einstein College of Medicine, Montefiore Health System, Bronx, New York; and Department of Population Health, Dell Medical School, The University of Texas at Austin. None dropped out.

At the 2-day convening, participants described their departments in detail and discussed a wide range of topics, including their departments’ scopes, core themes, areas of focus, organizational structures, funding, health care delivery system engagements, intraschool relationships, institutional expectations, challenges, and opportunities. Members of the University of Texas at Austin’s Design Institute for Health^9^ provided questions, facilitated sessions, took field notes, and obtained audio recordings of the discussion. Shared themes and organizational similarities and differences were elicited through a facilitated group consensus process and honed by participants over subsequent group emails and conference calls.^10^

The horizon scan was conducted by 1 of the chairs (M.N.G.) by manually searching the websites of all 149 accredited US medical schools that were active members of the Association of American Medical Colleges. For departments that had the word *population* in their names, the date of inception and academic structure were ascertained and confirmed by direct outreach to the department chair.

## Results

Eight of 9 population-oriented departments in the working group had come into being in the last 6 years (Table). The 9 departments had 5 to 97 full-time faculty. Despite varied organizational structures, all addressed some or most of a set of core areas of focus that spanned research, education, and service. Departments were diverse in their relationships with the delivery of clinical care at their institutions. All engaged in varied forms of community partnership. The eFigure in the Supplement depicts the range of areas of focus across mission areas and spanning academia, clinical care, and community, presently embodied by population health–oriented departments in academic medicine.

**Table.** Population-Focused Departments in Academic Medicine, 2018

Department Name	Institution	Year Founded	Structure and Organization	No. of Faculty^a^	Teaching Programs	Service^b^
Department of Population Health Sciences	University of Wisconsin School of Medicine and Public Health, Madison	1959, As Department of Preventive Medicine; name changed to current in 2001	Concentrations Epidemiology Health services research	23	MS programs Population health Epidemiology PhD programs Population health Epidemiology Global Health Certificate Preventive medicine residency	Survey of the Health of Wisconsin County Health Rankings and Roadmaps
Department of Epidemiology and Population Health	Albert Einstein College of Medicine, Bronx, New York	1955, As Department of Preventive and Environmental Medicine; name changed in 1960s to Department of Community Health; named changed to current in 2003	Divisions Biomedical and Bioethics Research Training Biostatistics Community Collaboration and Implementation Science Epidemiology Health Promotion and Nutrition Research Sections and centers Epidemiology Informatics and Study Management Unit Montefiore Office of Community and Population Health NY Regional Center for Diabetes Translational Research Quantitative Sciences in Biomedical Research Center Montefiore Hudson Valley Collaborative	53	MS programs Bioethics (also a certificate program) Clinical and population health research methods PhD program in clinical investigation Montefiore-Einstein Certificate in Bioethics and Medical Humanities Principles of Preventive Medicine course for undergraduate medical students	Montefiore Office of Community and Population Health
Department of Population Medicine	Harvard Medical School, Boston, Massachusetts	1992, As Department of Ambulatory Care and Prevention; name changed to current in 2009.	Divisions Therapeutics and Infectious Disease Epidemiology Chronic Disease Research Across the Lifecourse Health Policy and Insurance Research Child Health Research Programs Biostatistics Centers Center for Cancer Policy and Program Evaluation Precision Medicine Translational Research Center	46	Mandatory medical school courses in clinical epidemiology and population health Approximately 20 full-time trainees per year Harvard-wide fellowship programs General medicine Pediatric health services research Health policy Predoctoral and postdoctoral program trainees Shared primary care residency with Atrius Health and Brigham and Women’s Hospital	Led the MDPHnet Project on behalf of the Massachusetts Department of Public Health Led US Food and Drug Administration Sentinel System
Department of Medical and Population Health Sciences Research	Florida International University, Herbert Wertheim College of Medicine, Miami	2010	Divisions Medical Student Research and Learning Applied Health Sciences Research Faculty Support and Development Sections Biostatistics Data Management	7	Medical school courses Quantitative research methods Evidence-based medicine Research scholarship Support for graduate programs research activities	Led consecutive population-based surveys of local neighborhoods Supporting health disparities research and education
Department of Population Health	New York University School of Medicine, New York	2012	Divisions Health and Behavior Comparative Effectiveness and Decision Science Healthcare Delivery Science Epidemiology Biostatistics Medical Ethics Sections and centers Healthful Behavior Change Health Equity Tobacco, Alcohol and Drug Use Health Choice, Policy and Evaluation Early Childhood Health and Development Community Service Plan	97	4-y Medical school including 3-y pathway Longitudinal population health courses Health systems Health policy Population health MS programs Clinical investigation Systems and computational biomedicine Bioethics PhD programs Epidemiology Biostatistics Population health science (fall 2020) GME offerings in population health	Predictive health care delivery analytics to accelerate development of learning health system Disseminate results of delivery system innovation through scholarship and publication Responsible for Community Health Improvement component of institution’s Community Benefit efforts
Department of Population Health Science and Policy	Icahn School of Medicine at Mount Sinai, New York, New York	1995, As Department of Health Policy; name changed to current in 2014	Divisions and centers Center for Biostatistics International Center for Health Outcomes and Innovation Research Center for Health Equity and Community Engaged Research Center for Behavioral Oncology Division of Health Policy and Economics Affiliated institutes Institute for Healthcare Delivery Sciences Institute for Translational Epidemiology Women’s Health Research Institute	41	MS programs Healthcare delivery leadership Biostatistics Reach for Your First NIH K/R Award	Analytics support for Health System Biostatistics consultation service Community-Engaged Research Board NIH CTSA (KL2 training, research design and methods, patient and clinician interactions, and community engagement)
Department of Population Health Sciences	University of Utah School of Medicine, Salt Lake City	2014	Divisions Health Systems Innovation and Research Cancer Population Sciences Biostatistics	16	PhD program in population health sciences 4-y Pathway in population health (medical student elective)	NIH CTSA (TL1, biostatistics) Methodological expertise and infrastructure support for clinical research Tobacco cessation in FQHC Wellness Mobile Health Vehicle for diabetes prevention Developing engagement opportunities with nonprofit and community organizations, health departments, and health systems within the Mountain West
Department of Family, Population and Preventive Medicine	School of Medicine, Stony Brook University, Stony Brook, New York	2015	Divisions Epidemiology and Biostatistics Family and Community Medicine GME Medicine in Society Occupational, Environmental and Clinical Preventive Medicine Nutrition Preventive Medicine and Population Health Center Center for Medical Humanities, Compassionate Care and Bioethics	45	MA programs Medical humanities Compassionate care Bioethics Core faculty for MPH program Residency programs General preventive medicine Public health Family medicine	Biostatistics Consulting Core Major contributor to DSRIP initiative in Suffolk County Faculty involved in community outreach and physicians precept at student-run clinic
Department of Population Health	Dell Medical School, University of Texas at Austin	2016	Divisions Community Engagement and Health Equity Primary Care and Value-Based Health Occupational and Environment Medicine Health Information and Data Analytic Sciences Education and Training Global Health Sections and centers Division of Family and Community Medicine Center for Place-Based Initiatives Health Services and Community-Based Research Community Strategy Team	40	2-y Clerkship in primary care and community engagement Family medicine residency program Health equity training for medical school faculty and staff Educational exchange in global health settings in Kenya and Latin America 2-y Postdoctoral research fellowships for physicians and PhDs	Consolidated primary care into a single initiative across all medical school departments Neighborhood Health Initiative implementing Community-Centered Health Home model in specified neighborhoods and public housing complexes
Department of Population Health and Biostatistics	University of Texas Rio Grande Valley School of Medicine, Edinburg, Harlingen, and Brownsville	2016	Units Biostatiscs reseach and computing Community Health Partnerships Community Advisory Boards AHECs, including primary care services	5	AHEC Scholars Program Global health Communiry service learning	Regional safety net clinics consortium
Department of Population and Public Health Sciences	Boonshoft School of Medicine, Wright State University, Dayton, Ohio	1973, As Department of Community Health; name changed to current in 2016	Divisions Epidemiology and Biostatistics International and Environmental Health Social and Behavioral Determinants Health Systems and Policy Center Center for Investigation, Treatment and Addiction Research	15	Undergraduate global health course Medical school curriculum in ethics, population health, and prevention MPH program with concentration Population health Health promotion and education Graduate-level certificates Global health Health care management Public health leadership Epidemiology MBA program with health care concentration	Reach Out of Montgomery County Contracts and consultative services with regional health departments
Department of Population and Quantitative Health Sciences	Case Western Reserve University, Cleveland, Ohio	1987, As Department of Epidemiology and Biostatistics; name changed to current in 2017	Areas of focus Population health Biostatistics Genetic epidemiology Epidemiology Bioinformatics Health services research	34	MPH program MS programs Biostatistics Health informatics Clinical research PhD programs Epidemiology Biostatistics Health informatics	Prevention Research Center for Healthy Neighborhoods Biostatistics core Bioinformatics core Data management core
Department of Population Health Sciences	Duke School of Medicine, Durham, North Carolina	2017	Sections and centers Epidemiology Implementation science Health services research Health measurement	34	MS program in population health sciences (fall 2019) PhD program in population health sciences (fall 2021)	Shared data and analytics service Consultation on health measurement, stakeholder engagement, and empirical bioethics
Department of Population Health Sciences	Medical College of Georgia at Augusta University, Augusta	2017	Divisions Biostatistics and Data Science Epidemiology Georgia Prevention Institute	25	MS program in biostatistics PhD program in biostatistics Biostatistics and epidemiology courses in allied health and nursing	Research Support Center Community engagements to promote health behavior and public health
Department of Population Science and Policy	Southern Illinois University School of Medicine, Springfield	2018	Divisions Epidemiology and Biostatistics Human and Community Development Health System Science	7	4-y Medical student longitudinal curriculum MD/MPH program logistics and coursework	Consultation on design and implementation of population health–level programs Education workshops

Abbreviations: AHEC, Area Health Education Center; CTSA, Clinical and Translational Science Award; DSRIP, Delivery System Reform Incentive Payment; FQHC, Federally Qualified Health Center; GME, graduate medical education; NIH, National Institutes of Health.

^a^ Includes only full-time faculty with primary appointments.

^b^ Including delivery system support, community health needs assessment, and community benefit involvement.

While many of these areas of focus are not new in academic medicine, several essential themes emerged. First, many groups adopt an overarching focus on methods and the nature of population-level approaches, in contrast with academic medicine’s traditional focus on specific clinical conditions. Although initiatives involving specific conditions, such as diabetes, asthma, and colorectal cancer detection, may act as the catalyst, methods and strategies are typically given higher priority. Second, applied research is highly valued. In some cases, this takes the form of an emphasis on implementation and dissemination science, with a focus on real-world or frontline settings. All of the departments were committed to a wide range of partnerships within their local academic communities and with their institutions’ health care delivery systems (eg, fostering care transformation, innovation, and quality; and helping to support and accelerate development of a learning health care system). Third, all departments expressed a strong commitment to partnering with community members and organizations in planning and conducting research, including joint goal setting, developing trust, and advancing health equity. Fourth, the importance of cross-sectoral initiatives was widely acknowledged, bridging academic medicine not only with public health but also with other sectors with substantial health impact (eg, housing and education).

Reviewing the websites of accredited US medical schools, we identified 15 with department names that included the word *population*. (The 6 departments that were not part of the 9 participants in the initial convenings were the Department of Population and Quantitative Health Sciences, Case Western Reserve University School of Medicine, Cleveland, Ohio; Department of Medical and Population Health Sciences Research, Florida International University Herbert Wertheim College of Medicine, Miami; Department of Family, Population and Preventive Medicine, Stony Brook University of Medicine, Stony Brook, New York; Department of Population Health Sciences, Medical College of Georgia at Augusta University, Augusta; Department of Population and Public Health Sciences, Boonshoft School of Medicine, Wright State University, Dayton, Ohio; and Department of Population Science and Policy, Southern Illinois University School of Medicine, Springfield.) The Table contains descriptive data for the 15 departments identified as of November 2018. At some AMCs, departments with other names (eg, *Preventive Medicine*), launched decades ago, have more recently broadened their focus from patient-level disease prevention to more population-oriented approaches. However, such entities did not meet the search criterion of including *population* in their titles. Additionally, Jefferson University in Philadelphia, Pennsylvania, launched a College of Population Health in 2008. A leader in this field, the Jefferson University College of Population Health represents an effort at the university level, whereas we discuss the emergence of population health as a discipline in US medical schools. Schools of public health are grappling with how to incorporate growing attention to population health into their missions as well. Indeed, the Association of Schools and Programs in Public Health^11^ recently released the final report of its Population Health Initiative that wrestled with the evolution and differentiation of population health within its member institutions. While some of the population health–oriented departments identified existed within universities that also had schools of public health, no common patterns emerged regarding relationships between such departments and their sister schools of public health.

## Discussion

### Tensions

A number of challenges and tensions face the emergence of departments of population health in academic medicine. Boundaries with other departments’ areas of focus can become blurred. For example, a methods-oriented academic department of population health might build capacity that overlaps with that of a hospital’s business analytics unit, or another department might launch a community-based initiative to increase human papillomavirus vaccination. Administrative and organizational incentives are vital to the nourishment and alignment of such cross-departmental opportunities. Areas of overlapping focus can also arise when an AMC has a department of family and community medicine or a school of public health under the same university umbrella as a department of population health. Such entities often apply many of the same methods of and engage with similar partners to AMC-based departments of population health. Clear communication regarding principal areas of focus and complementary strengths can be valuable in structuring and bridging these boundaries to design, implement, test, and disseminate interventions for sustainable improvements in population health while avoiding academic silos and unnecessary competition.

The partnerships so vital to advancing meaningful population health research and action can take years to develop, and relationships between an AMC and its surrounding communities are often complex. Substantial investments in time, resources, and trust building are essential to the success of community-engaged partnership.

Building and sustaining population health research requires that traditional funding mechanisms (eg, from the National Institutes of Health [NIH]) be complemented by other federal and nonfederal sources, yet many medical schools value NIH grants most highly. While NIH funding for research in some areas relevant to population health has increased substantially in recent years (eg, implementation science, health disparities, and comparative effectiveness), truly expanding population health research requires institutional support for a mixed portfolio of extramural awards. Furthermore, competing successfully for institutional support for population health research can be challenging in the context of many schools’ longstanding focus on biomedical research and emerging priorities, like precision medicine. Measurable, long-term improvements in population health will require stable departments, institutes, and centers that are incentivized by their AMCs to develop broad and sustainable portfolios of funding sources, including federal research and service grants; city, county, and state public health contracts; philanthropy; and mutually beneficial initiatives with the private sector.

Academic promotion and tenure policies must evolve to recognize and reward faculty who make important scientific contributions to population health research and are able to grow and sustain their research through a variety of funding mechanisms. Further, AMCs’ service mission must transcend clinical programs to include programs and interventions aimed at overcoming social barriers to health as legitimate accomplishments for promotion and tenure. Measures of success also need to recognize the time required for population health investigators to design and conduct their research and affect population-level outcomes.

Engaged partnerships between clinical/operational leaders and faculty researchers are vital if academic departments of population health are to contribute meaningfully to learning health systems at their home institutions. Such partnerships require that researchers understand the operational pressures and timelines faced by the delivery system and that clinical leaders value the role of rigorous science in generating reliable, actionable evidence.

### Opportunities

Firmly establishing academic medicine’s commitment to the principles of population health—especially promoting health, preventing disease, and eliminating health inequities—and to building and advancing related research, training, and community-level applications represents a major step forward in bridging medicine and public health and holds great promise. One important opportunity to advance population health within US medical schools is promoting a holistic view of health that includes both clinical and social determinants of health, well-being, disease, and disability and the multidisciplinary and cross-sector interventions and policies required to address them, such as early childhood education, economic development, and environmental protection.^12^ This would help extend the engagement of health care beyond its principal focus on sick care (ie, diagnosis and treatment) to encompass both traditional and upstream approaches to prevention.

Engaging community residents and leaders as equal partners in health improvement, including generating ideas to overcome local barriers to progress, is another opportunity to advance population health. This requires information sharing, mutual trust and respect, intensive listening, and understanding the history of experiences each party brings to the conversation. Community benefit–spending can provide resources to strengthen frontline partnerships. Medical schools can also help by supporting health care delivery systems in addressing high levels of social need, including those of high-cost patients, in part by facilitating engagement with community resources. Another key opportunity for medical schools to advance population health is by reinvigorating institutional acceptance of a social justice mission as integral to health care delivery and by training the next generation of scholars to solve pressing challenges of improving population health and advancing health equity.

### Consolidating Gains and Facilitating Progress

The group articulated 3 steps important to consolidating gains made by early population health–oriented departments and facilitating the further growth of the field. There was interest in creating an organizing forum to share approaches to developing and supporting such departments in AMCs. The Interdisciplinary Association of Population Health Science^13^ was cited as an example of a new organization with strong thematic alignment. In addition, although this group of leaders was drawn from academic medicine, there was strong consensus that a broad spectrum of disciplines is necessary to advance the science and practice of population health and that participation of analogous departments and centers in schools of public health and other entities will be essential to the field’s success going forward. University-level leadership can contribute to this objective.^14,15,16^ Finally, there was recognition that the growth of population health–oriented departments will be given further impetus by the growing movement for health care institutions to engage meaningfully with upstream determinants of health in the communities they serve.^12,17^ These departments are poised to lead in the quest for successful and economically sustainable paradigms for such efforts, including developing replicable platforms and approaches for engaging communities in true partnerships.

### Limitations

Our study had limitations. Our sample of leaders and horizon scan were limited to departments with the word *population* in their names. Entities with similar missions and interests were thereby excluded from our analysis. While this served our purpose in drawing attention to an important and growing trend, we recognize that other departments in academic medicine not covered here are concerned with related themes. Similarly, a variety of nondepartmental entities—centers, programs, institutes, and even schools—are emerging that address population health, including with the word *population* in their names. We limited our analysis to full departments to maintain thematic focus among the tensions and opportunities encountered in advancing these new initiatives. Furthermore, with new population health–oriented departments emerging at a rapid rate, some that are in planning stages or recently launched may not have been included in our tabulation. Additionally, it is too early to know which of the many approaches to studying, teaching about, and working to improve population health may be most effective and have enduring impact.

## Conclusions

Population health–oriented departments have emerged in academic medicine in recent years, reflecting growing recognition of the importance of their core areas of focus across the broad missions of research, education, and clinical care. In supporting this new framing, academic medicine affirms and strengthens its commitment to advancing population health and health equity, to improving the quality and effectiveness of care, and to upholding the social mission of medicine.

## Notes

Supplement. **eFigure.** Population Health–Focused Departments: Promoting Health, Preventing Disease, and Eliminating Health Inequities
