# Supplementary material for: The Emergence of Population Health in US Academic Medicine: A Qualitative Assessment
Source: JAMA Netw Open. 2019 Apr 12;2(4):e192200. doi: 10.1001/jamanetworkopen.2019.2200 (PMC6481447; doi:10.1001/jamanetworkopen.2019.2200)
Supplement: Supplement. — eFigure. Population Health–Focused Departments: Promoting Health, Preventing Disease, and Eliminating Health Inequities [file jamanetwopen-2-e192200-s001.pdf]

Supplementary Online Content

Gourevitch MN, Curtis LH, Durkin MS, et al. The emergence of population health in US academic medicine: a qualitative assessment. *JAMA Netw Open*. 2019;2(4):e192200. doi:10.1001/jamanetworkopen.2019.2200

eFigure. Population Health–Focused Departments: Promoting Health, Preventing Disease, and Eliminating Health Inequities

This supplementary material has been provided by the authors to give readers additional information about their work.

© 2019 Gourevitch MN et al. *JAMA Network Open.*

eFigure. Population Health–Focused Departments: Promoting Health, Preventing Disease, and Eliminating Health Inequities

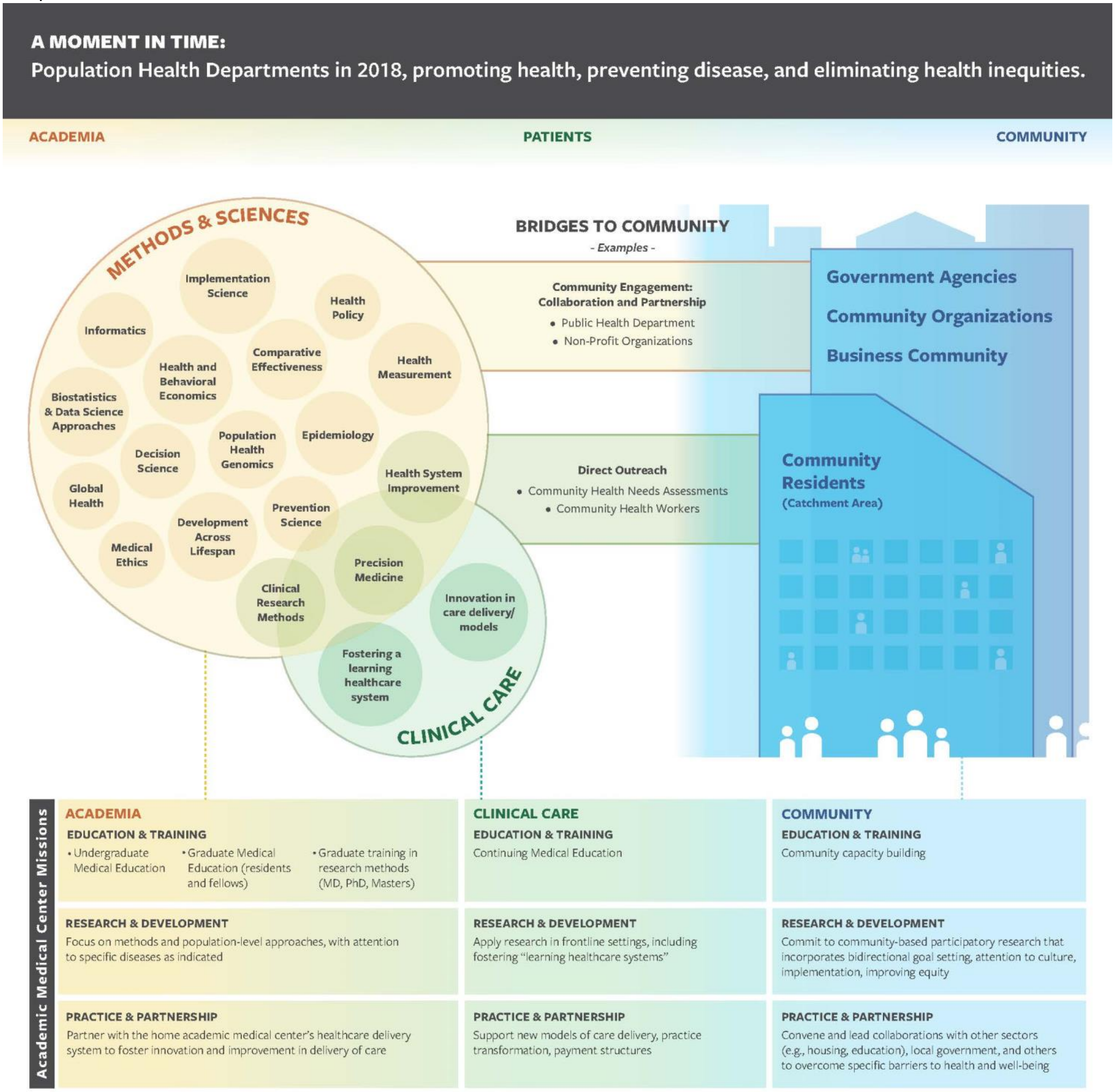

© 2019 Gourevitch MN et al. *JAMA Network Open.*
